# Goat Milk Supplementation Modulates the Mitochondrial Metabolic Flexibility and Orexin-A Levels Influencing the Inflammatory Pattern in Rats

**DOI:** 10.3390/antiox13091054

**Published:** 2024-08-29

**Authors:** Lidia Petrella, Rita Polito, Angela Catapano, Antonella Santillo, Maria Giovanna Ciliberti, Agostino Sevi, Antonietta Messina, Gina Cavaliere, Francesca Marino, Maria Grazia Polverino, Giovanni Messina, Marcellino Monda, Maria Pina Mollica, Marianna Crispino, Fabiano Cimmino, Marzia Albenzio, Giovanna Trinchese

**Affiliations:** 1 Department of Biology, University of Naples Federico II, 80126 Naples, Italy; lidia.petrella@unina.it (L.P.); angela.catapano@unina.it (A.C.); mariagraziapolverino.bio@outlook.it (M.G.P.); mariapina.mollica@unina.it (M.P.M.); fabiano.cimmino@unina.it (F.C.); giovanna.trinchese@unina.it (G.T.); 2 Department of Clinical and Experimental Medicine, University of Foggia, 71100 Foggia, Italy; rita.polito@unifg.it; 3 Department of Agriculture, Food, Natural Resources and Engineering (DAFNE), University of Foggia, 71100 Foggia, Italy; antonella.santillo@unifg.it (A.S.); maria.ciliberti@unifg.it (M.G.C.); agostino.sevi@unifg.it (A.S.); 4 Department of Precision Medicine, University of Campania, Luigi Vanvitelli, 80131 Naples, Italy; antonietta.messina@unicampania.it; 5 Department of Pharmaceutical Sciences, University of Perugia, 06126 Perugia, Italy; gina.cavaliere@unipg.it; 6 Department of Clinical Medicine and Surgery, University of Naples Federico II, 80131 Naples, Italy; francesca.marino2@gmail.com; 7 Department of Experimental Medicine, University of Campania Luigi Vanvitelli, 80131 Naples, Italy; giovanni.messina@unicampania.it (G.M.); marcellino.monda@unicampania.it (M.M.)

**Keywords:** nutrition, mitochondrial functions, goat milk, anti-inflammatory properties, antioxidant properties, orexin-A

## Abstract

Milk and its derivatives are included in a balanced diet of humans as excellent sources of proteins, vitamins, and essential minerals that are functional nutrients. Knowledge about the nutritional benefits or harms due to milk consumption has been expanding in recent years. We previously explored, in rodent models, the metabolic effects of isoenergetic intake of milk derived from cows, donkeys, or humans, while the impact of goat’s milk intake has remained unexplored. The aim of this work was to investigate, in an animal model, the effects of dietary supplementation with goat’s milk on energy homeostasis and inflammatory state, focusing on the modulation of mitochondrial functions in most metabolically active organs, such as skeletal muscle and the liver. In addition, we highlighted a link between nutrient intake, substrate metabolism, and the orexinergic system. Our results indicate that goat milk improves mitochondrial oxidative capacity and reduces inflammation and oxidative stress in both organs. Notably, goat milk lowers the circulating levels of Orexin-A, a neuropeptide that plays a crucial role in regulating peripheral energy balance and central nervous system mechanisms. These data provide the first evidence that the anti-inflammatory and antioxidant effects of goat milk are mediated by the modulation of mitochondrial functions and orexinergic signaling.

## 1. Introduction

Dairy and milk products are frequently included as important elements in a healthy and balanced diet [1,2]. Milk is the first food for mammals and provides the energy and nutrients necessary to ensure proper growth and development in the postnatal period, being crucial for bone mass formation [3,4,5]. Milk consumption generally stops at the end of the weaning period, except in humans, as it is consumed even during adulthood. However, several controversies arise from the consumption of dairy and milk products during adulthood, especially because they refer to milk derived from other species [6,7]. Despite these controversies, epidemiologic studies confirm the nutritional importance of milk in the human diet [1] and reinforce its possible role in preventing several chronic conditions, the so-called Western diseases, like cardiovascular diseases, diabetes, obesity, and some forms of cancer [8].

The nutritional value of milk is unquestionable: it is a good source of proteins with key roles in immune function, as well as nutrient transport and absorption of important vitamins and essential minerals [1,9]. The chemical composition of milk can be influenced by several factors such as genetic profile of the animal species, animal environmental conditions, lactation stage, health [10] and mammalian gland status of the animals and their nutritional status [11,12,13]. Although cow milk is the most frequently consumed milk, many studies are being carried out on different types of milk produced from other animal species. Indeed, cow’s milk is often responsible for allergy onset and has been the object of criticism due to the quality of the lipid content and ethical problems related to intensive farming. Several studies have identified donkey milk, if adequately supplemented, as a suitable alternative to cow’s milk in children with cow’s milk allergies. Indeed, donkey milk has nutritional similarities to human milk and excellent palatability [14,15]. However, the high processing costs and limited production do not promote the widespread use of donkey milk in the adult population [16]. Thus, it is important to investigate valid alternatives for consumers.

Potential health benefits from the consumption of goat milk (GM) have recently been emphasized, regarding its hypoallergenicity, ability to limit the symptoms of gastrointestinal disorders, and improvement of Fe and Cu absorption, growth rates, bone density, and blood levels of vitamin A, Ca, thiamine, riboflavin, niacin, and cholesterol [17,18].

In comparative reviews, Park [19] and Raynal et al. [20] reported that goat milk has higher contents of A, B1, and B12 vitamins as well as calcium and phosphorus compared to cow and sheep milk. Compared with cow milk, goat milk forms softer curds in the stomach and contains a higher proportion of butterfat globules. Furthermore, the amino acid sequence and structure of the proteins in cow milk and goat milk are different, and these proteins are differently digested by humans. Numerous studies have compared the nutritional content and physiological properties of cow milk compared to goat milk [21]. In a comparative study of the features of cow’s and goat’s milk, Stergiadis et al. [18] showed a number of nutritionally desirable characteristics in goat’s milk, including lower concentrations of individual saturated fatty acids, which are associated with increased risk of cardiovascular diseases (C12:0, C14:0, C16:0), higher concentrations of omega-3 polyunsaturated fatty acid (PUFA), eicosapentaenoic acid (EPA), and docosahexaenoic acid (DHA) with positive effects on human health, an improved Na:K ratio, and high content of isoflavones (B, Cu, Mg, Mn, P) and minerals (Ca, S and Zn), which play major metabolic roles in the human body [18]. The differences in composition between cow and goat milk may be potentially explained by husbandry practices, grazing intake, forage/concentrate ratio, and mineral supplementation, which are different between cow and goat dairy production systems [18]. Also, different digestion behaviors between species and metabolic states of animals during diverse phases of lactation may have also contributed to this difference [22]. Although several compositional differences have been identified that also influence the nutrient intake of consumers, the implications of these differences on human health are not known [18]. Moreover, the link between nutrient intake, energy homeostasis, substrate metabolism, and the orexinergic system has been highlighted [23,24,25]. In particular, Orexin-A, also known as hypocretin-1, is a multifunctional neuropeptide produced by specific neurons of the lateral hypothalamus, which plays a pivotal role in regulating metabolism. Its effects on energy expenditure, feeding behavior, and interaction with other metabolic hormones highlight its importance in maintaining energy balance and metabolic homeostasis [26].

Based on this background, and considering the multiple mechanisms that regulate the nutritional status following the intake of nutrients, we decided to investigate the effects of dietary supplementation with goat’s milk on an animal model, evaluating its metabolic effects on body composition, mitochondrial function, and efficiency, as well as inflammatory parameters. We have investigated the metabolic and inflammatory profile in the main organs that are regulators of energy expenditure and lipid and glucose metabolism, such as the liver and skeletal muscle, and we explored a potential influence on orexin levels.

## 2. Materials and Methods

### 2.1. Animals and Chemicals

All chemicals were purchased from Sigma–Aldrich (St. Louis, MO, USA), unless specified otherwise. Male Wistar rats (60 days old; ≈350 g; Charles River, Calco, Lecco, Italy) were caged in a temperature-controlled room and exposed to a daily light–dark cycle (12/12 h) with free access to standard chow diet (15.88 kJ/g) and drinking water. After a week of acclimatization, the rats were divided into two experimental groups (n = 8 each), one that kept eating only standard chow diet (CTR) and the other received, in addition to the diet, a daily intake of raw goat milk (GM, 82 kj, 21 mL/day), at an energetic dose used in previous experiments with milk-fed rats, for 4 weeks [27]. GM was obtained from a dairy farm located in the province of Foggia (Apulia region, Italy). The flock, consisting of about 200 mid-lactating Garganica goats, grazed in fenced paddocks in the morning and was housed in shaded open pens at night. The pasture composition consisted predominantly of Graminaceae, and also of Papilionaceae, Compositae, and Leguminosae, and animals were supplemented with oat hay, and oat and barley grains. Lactating goats were in parity 1–6 and were machine-milked twice daily with an average milk yield of about 780 g/day. Before milk collection, goats were examined by a veterinarian to confirm the absence of any sign of clinical mastitis. Bulk milk from the morning milking was sampled, immediately transported to the laboratory under refrigeration, and analyzed for gross composition (Milko Scan 133B; Foss Electric, Hillerød, Denmark), pH (GLP 21 Crison, Spain), and somatic cell count (Fossomatic Minor; Foss Electric, Hillerød, Denmark). Mean values were 4.3 ± 0.09%, 4.5 ± 0.07%, and 3.4 ± 0.06%, 2.5 ± 0.06% for fat, lactose, protein, and casein, respectively; 6.63 ± 0.06 for pH; and 383 ± 57 cells × 10^3^/mL for milk somatic cells. Milk was heat-treated in a stainless-steel container, double jacketed with an internal propeller (CASARO, Philips, Netherlands) at 75 °C for 15 s and immediately cooled down in an ice bath (0 ± 2 °C) and frozen for further use. Standard rodent diet (4RF21) was purchased from Mucedola (Mucedola srl, Settimo Milanese, Milan, Italy). At the end of the treatments, the animals were anesthetized by intra-peritoneal injection of chloral hydrate (40 mg/100 g body weight), and blood was taken from the inferior cava vein. The liver and skeletal muscle were removed and immediately processed to obtain the mitochondrial fraction, and the samples not immediately used for mitochondrial preparation were stored at −80 °C. Animal experiments were carried out following the Directive 2010/63/UE, enforced by Italian D.L. 26/2014, and approved by the animal care and the Committee of the University of Naples ”Federico II” (OPBA), Naples, Italy, and the Italian Ministry of Health, Rome, Italy (authorization n. 97/2019-PR).

### 2.2. Body Composition and Energy Balance

During the experimental period, body weight and food intake were monitored daily to calculate body weight gain and gross energy intake. Body composition and energy balance assessments were carried out immediately after animals were sacrificed by comparative carcass evaluation as previously reported [28].

### 2.3. Mitochondrial Preparation and Analysis

The liver was finely chopped and washed in a solution medium containing 220 mM-mannitol, 70 mM sucrose, 20 mM HEPES (pH 7.4), 1 mM-EDTA, and 0.1% (*w*/*v*) fatty-acid-free bovine serum albumin (BSA). Tissue samples were homogenized with medium (1:4, *w*/*v*) in a Potter Elvehjem homogenizer (Heidolph, Kelheim, Germany) set at 500 rpm (4 strokes/min). The homogenate was centrifuged (1000× *g*, 10 min, 4 °C) and the resulting supernatant fraction was again centrifuged (3000× *g*, 10 min, 4 °C). The mitochondrial pellet was washed twice and finally resuspended in a medium containing 80 mM-KCl, 50 mM-HEPES (pH 7.0), 5 mM KH_2_PO_4_, and 0.1% (*w*/*v*) fatty-acid-free BSA.

Limb leg muscle aliquots were stripped of excess fat and connective tissue, finely chopped, and washed in a buffered solution (100 mM KCl, 50 mM Tris-HCl, pH 7.5, 5 mM MgCl_2_, 1 mM EDTA, 5 mM EGTA, 0.1% (*w*/*v*) fatty-acid-free bovine serum albumin (BSA)). Tissue fragments were homogenized with the above buffer (1: 8, *w*/*v*) in a Potter Elvehjem homogenizer (Heidolph, Kelheim, Germany) set at 500 rpm (4 strokes/min), filtered by sterile gauze and finally centrifuged (3000× *g*, 10 min, 4 °C). The pellet was resuspended and centrifuged at 500× *g* for 10 min. The supernatant was further centrifuged (3000× *g*, 10 min, 4 °C) and the pellet, containing the mitochondrial fraction, was dissolved in suspension medium [29].

The Hartree method using BSA as the protein standard was used to determine the protein content of the mitochondrial fraction [30]. The mitochondrial O_2_ consumption rate was estimated by a Clark-type electrode (Yellow Springs Instruments, Yellow Springs, OH, USA) in a water-jacketed chamber at 30 °C. Hepatic and skeletal muscle mitochondria (0.5 mg protein) were incubated in a buffer containing 80 mM KCl, 50 mM HEPES, 1 mM EGTA, 5 mM KH_2_PO_4_ (pH 7.0), and 0.1% (*w*/*v*) fatty-acid-free BSA. The substrates used for the determination of hepatic and muscle mitochondrial respiratory rates were 10 mM succinate + 3.75 μM rotenone or 40 μM palmitoyl-carnitine + 2.5 mM-malate, the latter used for the determination of fatty acid oxidation rate. State 3 of mitochondrial respiration was determined in the presence of 0.6 mM ADP. The ratio between states 3 and 4, called the respiratory control ratio, was calculated according to Estabrook. The addition of ADP after the substrate to the mitochondrial incubation allows the ATP synthase to function and the electron transport chain to accelerate (‘state 3ADP’). When the ATP/ADP ratio approaches equilibrium, proton re-entry through the ATP synthase stops and respiration slows (‘state 4′). In control experiments, we assessed the purity of mitochondrial preparation by checking possible contamination by other ATPase-containing membranes <10%, whereas the quality of mitochondrial preparation was assessed by adding cytochrome c (3 nmol/mg protein) and evaluating an enhancement in state 3 respiration rate ≤10%, as previously indicated. The degree of coupling was determined in the liver by applying the equation by Cairns et al. [31]:

$$\mathrm{degree}\;\mathrm{of}\;\mathrm{coupling}=\sqrt{1-\left(Jo\right)sh/\left(Jo\right)unc}$$

where (Jo)sh represents the oxygen consumption rate in the presence of oligomycin that inhibits ATP synthase, and (Jo)unc is the uncoupled rate of oxygen consumption induced by carbonyl-cyanide-p-trifluoromethoxyphenylhydrazone (FCCP), which dissipates the transmitochondrial proton gradient. (Jo)sh and (Jo)unc were measured as above using succinate (10 mmol/L) and rotenone (3.75 µmol/L) in the presence of oligomycin (2 µg/mL) or FCCP (1 µmol/L), respectively. Mitochondrial H_2_O_2_ yield was assayed by following the linear increase in fluorescence (ex 312 nm and em 420 nm) due to the oxidation of homovanillic acid in the presence of horseradish peroxidase [32].

### 2.4. Evaluation of Inflammatory Markers

Commercially available ELISA kits were used to determine tumor necrosis factor-alpha (TNF-α), interleukin-6 (IL-6), and interleukin-1 β (IL-1 β) (Thermo Scientific, Rockford, IL, USA; Biovendor R and D, Brno, Czech Republic) concentrations in liver and skeletal muscle homogenates [33].

### 2.5. Evaluation of Redox Status

The dithionitrobenzoic acid–GSSG reductase recycling assay was used to measure the concentrations of reduced glutathione (GSH) and oxidized glutathione (GSSG) in the liver and skeletal muscle homogenates of differently treated rats [34,35]. Upon normalization to the protein content, GSH and GSSG amounts were finally expressed as nmoles/mg prot/min.

The thiobarbituric acid reaction (TBAR) method was used in order to determine the level of malondialdehyde (MDA) in the liver and skeletal muscle homogenates. At a wavelength of 532 nm, MDA and thiobarbituric acid (TBA) combine to generate a pink chromogen, indicative of lipid peroxidation. MDA values were expressed as nanomoles per milligram of protein [36].

### 2.6. Evaluation of Orexin-A Serum Concentration

A sandwich ELISA assay was used to measure Orexin-A concentration in rat serum samples. The concentration of rat OXA in the samples was determined by comparing the OD of the samples to the standard curve, and it was measured spectrophotometrically at a wavelength of 450 nm ± 10 nm, according to the manufacturer’s instructions (Rat OXA Elisa kit, ELK Biotechnology, Wuhan, China). Each sample was tested three times in duplicate.

### 2.7. Statistical Analyses

All values were reported as means ± SEM. Data, significantly different from controls by Student’s unpaired *t*-test (*p* < 0.05), are from n = 8 animals/group. * *p* < 0.05; ** *p* < 0.01; *** *p* < 0.001; and **** *p* < 0.0001 indicate statistically significant differences. All analyses were executed with GraphPad Prism 8 (GraphPad Software, San Diego, CA, USA).

## 3. Results

### 3.1. Goat Milk Administration Modulates Body Weight Gain with No Changes in Body Composition

**Figure 1:**
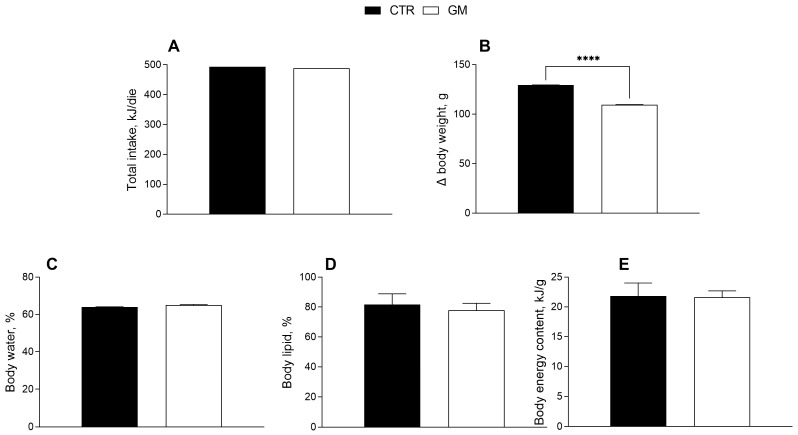
Metabolic profile in control and goat milk-treated rats. Daily food intake at the end of treatment (**A**), body weight (**B**), body water (**C**), lipid (**D**) and energy content (**E**) are reported. Data are presented as means ± SEM from n = 8 animals/group. **** *p* < 0.0001 goat milk (GM) vs. control (CTR).

The effects of goat milk supplementation on the body weight and composition of rats are shown in Figure 1. Body weight gain in the GM group was significantly lower than in the control group (Figure 1B), although the total caloric intake (including the calories from milk supplementation) was not significantly different (Table 1, Figure 1A). No significant difference was shown for body energy and lipids and water content percentage between the two groups (Figure 1C–E).

### 3.2. Modulation of Liver and Skeletal Muscle Inflammatory Profile in Goat Milk-Supplemented Rats

**Figure 2:**
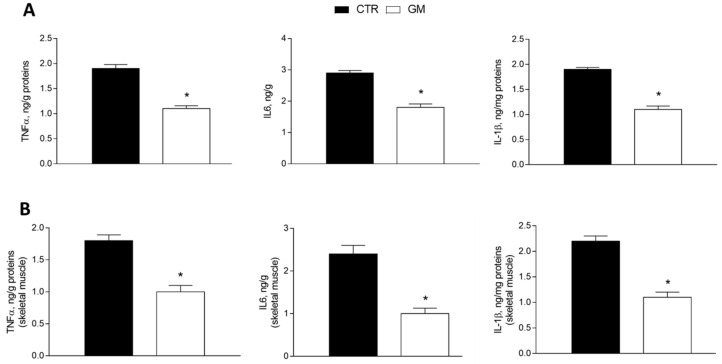
Inflammatory markers (TNF-α, IL6, and IL1β) in the liver (**A**) and skeletal muscle (**B**) of control and goat milk-treated rats. Data are presented as means ± SEM from n = 8 animals/group. * *p* < 0.05 goat milk (GM) vs. control (CTR).

The levels of proinflammatory markers (tumor necrosis factor, TNF-α; interleukin-6, IL-6; and interleukin-1β, IL-1β) were significantly reduced in the liver homogenate from GM-supplemented rats compared to the control group (Figure 2A). The same effect was observed in the skeletal muscle homogenate of the GM group, which displays lower levels of inflammatory cytokines compared to the control group (Figure 2B).

### 3.3. Liver and Skeletal Muscle Mitochondrial Oxidative Capacity Is Modulated by the Administration of Goat Milk

**Figure 3:**
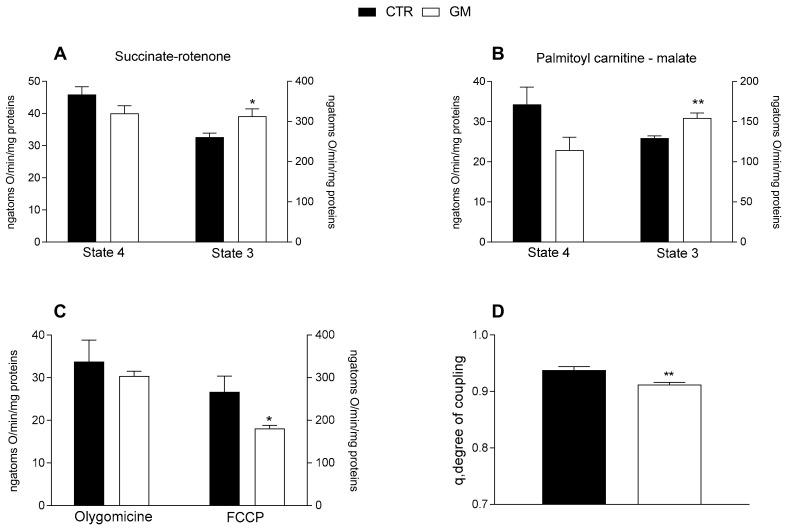
Hepatic mitochondrial functions in control and goat milk-treated rats. Hepatic mitochondrial respiration rates were evaluated with succinate + rotenone (**A**), or palmitoyl-carnitine + malate (**B**) as substrates. Mitochondrial oxygen consumption in the presence of oligomycin and uncoupled by carbonyl-cyanide-4(trifluoromethoxy)phenylhydrazone (FCCP) (**C**), and the degree of coupling (**D**) are shown. Data are presented as means ± SEM from n = 8 animals/group. * *p* < 0.05, ** *p* < 0.01 goat milk (GM) vs. control (CTR).

Mitochondrial state 3 respiration, evaluated using succinate or palmitoyl-carnitine as substrates (to detect fatty acid oxidation), of mitochondria isolated from the liver of GM-treated rats was increased compared with the control group (Figure 3A,B). No difference was observed in the oxygen consumption rate among the two groups after the addition of olygomicin, but a decrease in the GM-treated group was observed when the uncoupler FCCP was added (Figure 3C). Consequently, a lower mitochondrial coupling degree was observed in the GM-treated group than in control rats (Figure 3D).

**Figure 4:**
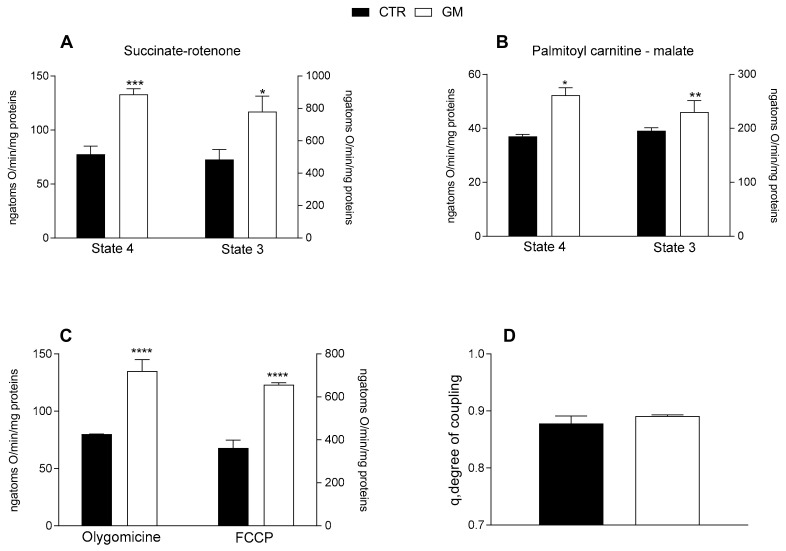
Skeletal muscle mitochondrial function in control and goat milk-treated rats. Skeletal muscle mitochondrial respiration rates were evaluated with succinate + rotenone (**A**), or palmitoyl-carnitine + malate (**B**) as substrates. Mitochondrial oxygen consumption in the presence of oligomycin and uncoupled by carbonyl-cyanide-4(trifluoromethoxy)phenylhydrazone (FCCP) (**C**), and the degree of coupling (**D**) are shown. Data are presented as means ± SEM from n = 8 animals/group. * *p* < 0.05, ** *p* < 0.01, *** *p* < 0.001, **** *p* < 0.0001 goat milk (GM) vs. control (CTR).

Both mitochondrial state 4 and 3 respiration, evaluated using succinate or palmitoyl-carnitine as substrates (to detect fatty acid oxidation), of mitochondria isolated from skeletal muscle was increased in GM-treated rats compared with the control group (Figure 4A,B). An increased oxygen consumption rate in the GM-treated group was also observed when olygomicin and FCCP were added (Figure 4C). No difference in the mitochondrial coupling degree was observed among the two groups (Figure 4D). 

### 3.4. Oxidative Stress and Antioxidant/Detoxifying Defense Are Improved by Goat Milk in Both Liver and Skeletal Muscle

**Figure 5:**
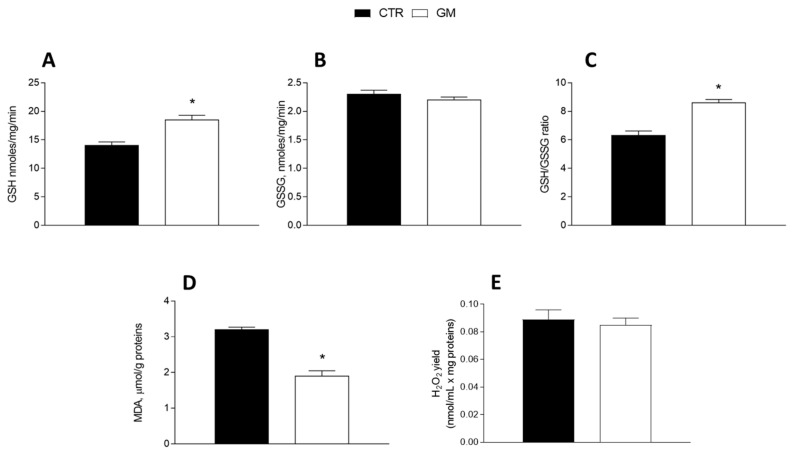
Hepatic oxidative stress profile in control and goat milk-treated rats. Reduced (GSH) and oxidized (GSSG) glutathione content (**A**,**B**), GSH/GSSG ratio (**C**), MDA levels (**D**), and H_2_O_2_ yield (**E**) were evaluated in hepatic tissue. Data are presented as means ± SEM from n = 8 animals/group. * *p* < 0.05 goat milk (GM) vs. control (CTR).

**Figure 6:**
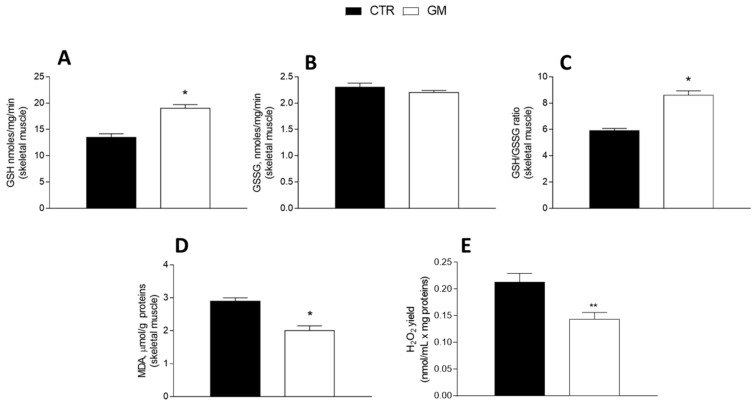
Skeletal muscle oxidative stress profile in control and goat milk-treated rats. Reduced (GSH) and oxidized (GSSG) glutathione content (**A**,**B**), GSH/GSSG ratio (**C**), MDA levels (**D**), and H_2_O_2_ yield (**E**) were evaluated in skeletal muscle tissue. Data are presented as means ± SEM from n = 8 animals/group. * *p* < 0.05, ** *p* < 0.01 goat milk (GM) vs. control (CTR).

GM treatment improved the antioxidant state in both the liver and skeletal muscle (Figure 5 and Figure 6). Indeed, while GSH levels significantly increased in GM-treated animals, compared to the control group (Figure 5A and Figure 6A), GSSG content did not change (Figure 5B and Figure 6B), resulting in an increased GSH/GSSG ratio in the GM group (Figure 5C and Figure 6C). In addition, malondialdehyde (MDA) levels also decreased in the GM group in both the liver and skeletal muscle (Figure 5D and Figure 6D). In isolated mitochondria, we also evaluated the H_2_O_2_ yield, an indirect index of ROS production, observing no variation in the mitochondria of hepatic tissue (Figure 5E), while a significant reduction in the release of H_2_O_2_ was observed in skeletal muscle (Figure 6E).

### 3.5. Orexin-A Serum Levels Are Strongly Modulated by Goat Milk Treatment

**Figure 7:**
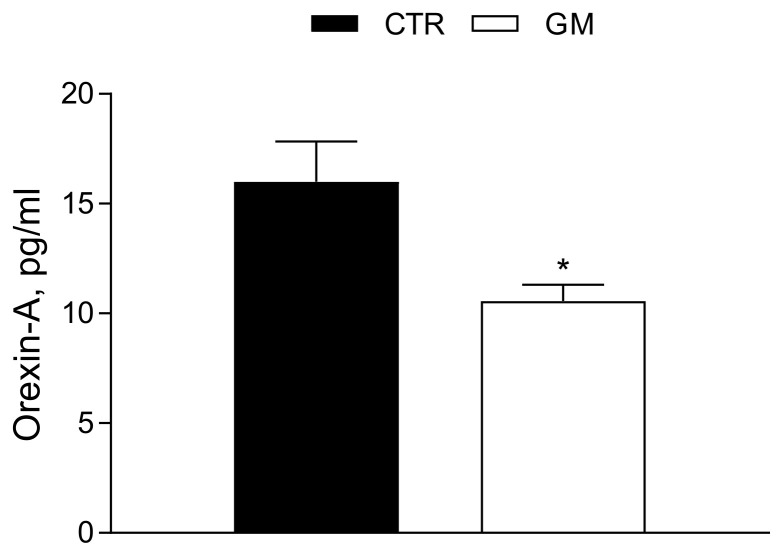
Orexin-A serum profile in control and goat milk-treated rats. The Orexin-A serum levels are strongly modulated by goat milk treatment compared to the control group. Data are presented as means ± SEM from n = 4 animals/group. * *p* < 0.05 goat milk (GM) vs. control (CTR).

The Orexin-A serum concentration was significantly reduced (Ctr 15.99 ± 1.84 pg/mL vs. GM 10.56 ± 0.75 pg/mL) in GM rats compared to the control group (see also Figure 7).

## 4. Discussion

In this study, we investigated the effects of dietary supplementation with goat’s milk (GM) on metabolic parameters in a rat model, focusing on its effects on liver and skeletal muscle. In particular, compared to untreated rats, GM-fed rats displayed a reduction in body weight gain, and an improvement in the inflammatory profile in both analyzed organs, associated with a modulation of mitochondrial functionality and oxidative stress. These results are particularly relevant considering the crucial role played by the liver and skeletal muscle in the metabolic homeostasis of the entire organism.

The decrease in body weight gain detected in GM-fed rats compared to untreated rats with the same daily calorie intake suggests that GM led to increased mitochondrial oxidation of energy substrates, especially in the organs most involved in energy expenditure, such as skeletal muscle. In fact, the evaluation of skeletal muscle mitochondrial function and efficiency following the administration of GM showed an improvement of the mitochondrial oxidative capacities both in the presence of glucidic substrates (succinate + rotenone) and lipid substrates (palmitoyl-carnitine + malate), considering the respiratory state 3 and state 4. These results indicate an improvement in skeletal muscle metabolic flexibility. Similarly, at the hepatic level, treatment with GM led to an improvement in mitochondrial oxidative capacities, especially at state 3, associated with reduced mitochondrial energy efficiency, linked to a major proton leak. Under these conditions, multiple substrates are burned to produce the same amount of adenosine triphosphate (ATP), creating a thermogenic effect. The improvement in mitochondrial functions found in GM-fed rats could be associated with the increase in omega-3 PUFA levels detected in goat milk. Indeed, it is well accepted that omega-3 PUFAs positively affect mitochondrial activity [37,38].

Since a low degree of inflammation is associated with chronic metabolic diseases related to unbalanced diet intake in our experiments, we analyzed the influence of GM in the modulation of inflammatory state. Therefore, we show that GM administration decreases TNFα, IL-6, and IL-1β levels in both liver and skeletal muscle. The decreased inflammation is also associated with a decrease in oxidative stress [38]. Thus, we analyzed the modulation of liver and skeletal muscle redox states following the administration of GM. We observed that GM-fed rats exhibited increased antioxidant capacity compared with control rats, with higher GSH levels, an increased GSH/GSSG ratio, and lowered levels of MDA, a marker of oxidative stress, in both the liver and skeletal muscle. These data can be explained by an improvement of substrate oxidation and decreased energy efficiency observed at the mitochondrial level. The reduced levels of MDA, an indicator of lipid peroxidation, are particularly relevant since lipid peroxidation is the first step on the way to the development of several chronic conditions, like insulin resistance and its associated diseases [39,40].

Additionally, we found lower levels of Orexin-A in the rats treated with GM compared to the control. Although this finding may seem contradictory to the reported increase in metabolic rate and reduction in inflammation, we believe that GM, being richer in fats, could induce an increase in the lipid droplets within adipose cells without increasing the animals’ body weight. Orexin-A, also known as hypocretin-1, is a neuropeptide produced by specific neurons in the lateral hypothalamus [41,42]. During fasting stress, Orexin-A plays a crucial role in regulating peripheral energy balance and central nervous system mechanisms, helping to coordinate sleep–wake cycles and motivated behaviors like food-seeking [43]. This neuropeptide exerts its effects by binding to the orexin type 1 receptor (OX1R), which is present in various tissues, including the intestine, pancreas, adrenals, kidneys, adipose tissue, and reproductive organs [44]. Orexin-A is considered a multifunctional molecule that regulates several vital body functions, including sleep/wake states, eating behavior, reward systems, energy homeostasis, cognition, and mood. Dysfunction in the orexinergic system is linked to various pathological conditions [45]. Moreover, this neuropeptide is crucial for energy balance and obesity management, influencing lipolysis in white fat and thermogenesis in brown fat, thus affecting overall energy balance [46,47]. Indeed, the hypothalamus is a critical brain region that plays a central role in regulating food intake and energy balance [48]. This regulation is complex and involves various hypothalamus nuclei, such as the arcuate nucleus (ARC), the paraventricular nucleus, the lateral hypothalamus (LH), and the ventromedial hypothalamus (VMH), as well as different neuronal circuits, such as the orexigenic neurons promoting food intake expressing neuropeptide Y (NPY) and agouti-related peptide (AgRP), and the anorexigenic neurons suppressing food intake expressing pro-opiomelanocortin (POMC) and cocaine- and amphetamine-regulated transcript (CART) [49,50]. The LH, known as a feeding center, produces orexins (hypocretins), which stimulate appetite and wakefulness [51]. Orexin-A, in particular, has a strong orexigenic effect. The VMH, often referred to as the satiety center, helps in signaling when to stop eating and is involved in regulating energy expenditure. Among key hormones and signals, leptin is produced by adipose tissue and signals satiety to the hypothalamus, particularly affecting the ARC. It inhibits NPY/AgRP neurons and stimulates POMC/CART neurons, thereby reducing food intake and increasing energy expenditure. Thus, there is negative feedback that helps prevent overeating by increasing anorexigenic signaling (i.e., leptin) and decreasing orexigenic signaling (i.e., Orexin-A) when energy stores are sufficient. This, in turn, could lead to higher leptin levels. It is well known that leptin and Orexin-A, through a negative feedback mechanism, regulate caloric intake [52]. Indeed, Orexin-A has an orexigenic effect, while leptin has an anorexigenic effect. High lipid intake affects both Orexin-A and leptin levels and their interactions, with several metabolic implications. Chronic high-fat diets can lead to leptin resistance, diminishing leptin’s ability to regulate appetite and energy expenditure effectively [53]. In this study, rats receiving goat milk supplementation, despite the higher intake of fatty acids, displayed a reduction in body weight without significant alterations in body lipid and energy content, probably due to a tightly cross-linked interaction between leptin and orexin release. Furthermore, leptin resistance may disrupt the inhibitory feedback on Orexin-A, potentially leading to altered energy homeostasis and lipid metabolism. However, the exact response of Orexin-A in the context of leptin resistance and high-fat diets requires further investigation [54]. The combined effects of Orexin-A and leptin in response to high lipid intake can influence overall metabolic health, impacting factors such as body weight, lipid profiles, and inflammatory states [37,53]. This could explain the observed weight reduction and increased metabolic rate. Therefore, we propose further studying the effects of goat’s milk on the adipocytokine pathway, particularly focusing on leptin.

## 5. Conclusions

In conclusion, our study highlights that rat dietary supplementation with goat milk decreases inflammatory status, improves mitochondrial lipid oxidation in the liver and skeletal muscle, and decreases Orexin-A levels. These data provide the first evidence that the beneficial effects of goat milk on rat metabolism are mediated by the modulation of mitochondrial functions and orexinergic signaling.

## Figures and Tables

**Table 1:** Food and energy intake.

	Food Intake (g/day)	Milk Intake (mL/day)	Food Intake (kj/day)	Milk Intake (kj/day)	Total Intake (kj/day)
Control	31.08	//	493.63	//	493.63
Goat milk-treated	25.57	21	406.11	82	488.11

## Data Availability

Data contained within the article.

## References

[1] Pereira P.C. (2014). Milk Nutritional Composition and Its Role in Human Health. Nutrition.

[2] Kliem K.E., Givens D.I. (2011). Dairy Products in the Food Chain: Their Impact on Health. Annu. Rev. Food Sci. Technol..

[3] Yackobovitch-Gavan M., Phillip M., Gat-Yablonski G. (2017). How Milk and Its Proteins Affect Growth, Bone Health, and Weight. Horm. Res. Paediatr..

[4] Melnik B. (2015). Milk—A Nutrient System of Mammalian Evolution Promoting mTORC1-Dependent Translation. Int. J. Mol. Sci..

[5] Ilesanmi-Oyelere B.L., Kruger M.C. (2020). The Role of Milk Components, Pro-, Pre-, and Synbiotic Foods in Calcium Absorption and Bone Health Maintenance. Front. Nutr..

[6] Norris J.M., Pietropaolo M. (1999). Controversial Topics Series: Milk Proteins and Diabetes. J. Endocrinol. Investig..

[7] Marangoni F., Pellegrino L., Verduci E., Ghiselli A., Bernabei R., Calvani R., Cetin I., Giampietro M., Perticone F., Piretta L. (2019). Cow’s Milk Consumption and Health: A Health Professional’s Guide. J. Am. Coll. Nutr..

[8] Melnik B.C. (2009). Milk—The Promoter of Chronic Western Diseases. Med. Hypotheses.

[9] Rice B.H., Quann E.E., Miller G.D. (2013). Meeting and Exceeding Dairy Recommendations: Effects of Dairy Consumption on Nutrient Intakes and Risk of Chronic Disease. Nutr. Rev..

[10] Albenzio M., Santillo A., Caroprese M., Ciliberti M.G., Marino R., Sevi A. (2016). Effect of Stage of Lactation on the Immune Competence of Goat Mammary Gland. J. Dairy Sci..

[11] Caroli A.M., Chessa S., Erhardt G.J. (2009). Invited Review: Milk Protein Polymorphisms in Cattle: Effect on Animal Breeding and Human Nutrition. J. Dairy Sci..

[12] Kalač P., Samková E. (2010). The Effects of Feeding Various Forages on Fatty Acid Composition of Bovine Milk Fat: A Review. Czech J. Anim. Sci..

[13] Nagahata H., Fuse M., Umehara K., Gondaira S., Higuchi H., Hisaeda K., Kumano Y. (2022). Evaluation of the Health Status of Mammary Glands and Compositional Changes in Udder-half Milk Obtained from Dairy Goats for Milk Quality Management. Anim. Sci. J..

[14] Sarti L., Martini M., Brajon G., Barni S., Salari F., Altomonte I., Ragona G., Mori F., Pucci N., Muscas G. (2019). Donkey’s Milk in the Management of Children with Cow’s Milk Protein Allergy: Nutritional and Hygienic Aspects. Ital. J. Pediatr..

[15] Lionetti L., Cavaliere G., Bergamo P., Trinchese G., De Filippo C., Gifuni G., Gaita M., Pignalosa A., Donizzetti I., Putti R. (2012). Diet Supplementation with Donkey Milk Upregulates Liver Mitochondrial Uncoupling, Reduces Energy Efficiency and Improves Antioxidant and Antiinflammatory Defences in Rats. Mol. Nutr. Food Res..

[16] Cimmino F., Catapano A., Villano I., Di Maio G., Petrella L., Traina G., Pizzella A., Tudisco R., Cavaliere G. (2023). Invited Review: Human, Cow, and Donkey Milk Comparison: Focus on Metabolic Effects. J. Dairy Sci..

[17] Haenlein G.F.W. (2004). Goat Milk in Human Nutrition. Small Rumin. Res..

[18] Stergiadis S., Nørskov N.P., Purup S., Givens I., Lee M.R.F. (2019). Comparative Nutrient Profiling of Retail Goat and Cow Milk. Nutrients.

[19] Park Y.W., Juárez M., Ramos M., Haenlein G.F.W. (2007). Physico-Chemical Characteristics of Goat and Sheep Milk. Small Rumin. Res..

[20] Raynal-Ljutovac K., Lagriffoul G., Paccard P., Guillet I., Chilliard Y. (2008). Composition of Goat and Sheep Milk Products: An Update. Small Rumin. Res..

[21] Zhou S.J., Sullivan T., Gibson R.A., Lönnerdal B., Prosser C.G., Lowry D.J., Makrides M. (2014). Nutritional Adequacy of Goat Milk Infant Formulas for Term Infants: A Double-Blind Randomised Controlled Trial. Br. J. Nutr..

[22] Bernard L., Toral P.G., Chilliard Y. (2017). Comparison of Mammary Lipid Metabolism in Dairy Cows and Goats Fed Diets Supplemented with Starch, Plant Oil, or Fish Oil. J. Dairy Sci..

[23] Imperatore R., Palomba L., Cristino L. (2017). Role of Orexin-A in Hypertension and Obesity. Curr. Hypertens. Rep..

[24] Mavanji V., Pomonis B., Kotz C.M. (2022). Orexin, Serotonin, and Energy Balance. WIREs Mech. Dis..

[25] Goforth P.B., Myers M.G., Lawrence A.J., De Lecea L. (2016). Roles for Orexin/Hypocretin in the Control of Energy Balance and Metabolism. Behavioral Neuroscience of Orexin/Hypocretin.

[26] Mavanji V., Pomonis B.L., Shekels L., Kotz C.M. (2024). Interactions between Lateral Hypothalamic Orexin and Dorsal Raphe Circuitry in Energy Balance. Brain Sci..

[27] Trinchese G., Cavaliere G., Canani R.B., Matamoros S., Bergamo P., De Filippo C., Aceto S., Gaita M., Cerino P., Negri R. (2015). Human, Donkey and Cow Milk Differently Affects Energy Efficiency and Inflammatory State by Modulating Mitochondrial Function and Gut Microbiota. J. Nutr. Biochem..

[28] Mollica M.P., Mattace Raso G., Cavaliere G., Trinchese G., De Filippo C., Aceto S., Prisco M., Pirozzi C., Di Guida F., Lama A. (2017). Butyrate Regulates Liver Mitochondrial Function, Efficiency, and Dynamics in Insulin-Resistant Obese Mice. Diabetes.

[29] Trinchese G., Cavaliere G., De Filippo C., Aceto S., Prisco M., Chun J.T., Penna E., Negri R., Muredda L., Demurtas A. (2018). Human Milk and Donkey Milk, Compared to Cow Milk, Reduce Inflammatory Mediators and Modulate Glucose and Lipid Metabolism, Acting on Mitochondrial Function and Oleylethanolamide Levels in Rat Skeletal Muscle. Front. Physiol..

[30] Hartree E.F. (1972). Determination of Protein: A Modification of the Lowry Method That Gives a Linear Photometric Response. Anal. Biochem..

[31] Cairns C.B., Walther J., Harken A.H., Banerjee A. (1998). Mitochondrial Oxidative Phosphorylation Thermodynamic Efficiencies Reflect Physiological Organ Roles. Am. J. Physiol.-Regul. Integr. Comp. Physiol..

[32] Barja G. (1998). Mitochondrial Free Radical Production and Aging in Mammals and Birds. Ann. N. Y. Acad. Sci..

[33] Cimmino F., Catapano A., Trinchese G., Cavaliere G., Culurciello R., Fogliano C., Penna E., Lucci V., Crispino M., Avallone B. (2021). Dietary Micronutrient Management to Treat Mitochondrial Dysfunction in Diet-Induced Obese Mice. Int. J. Mol. Sci..

[34] Cavaliere G., Trinchese G., Penna E., Cimmino F., Pirozzi C., Lama A., Annunziata C., Catapano A., Mattace Raso G., Meli R. (2019). High-Fat Diet Induces Neuroinflammation and Mitochondrial Impairment in Mice Cerebral Cortex and Synaptic Fraction. Front. Cell. Neurosci..

[35] Bergamo P., Maurano F., Rossi M. (2007). Phase 2 Enzyme Induction by Conjugated Linoleic Acid Improves Lupus-Associated Oxidative Stress. Free Radic. Biol. Med..

[36] Lu H., Zhang D.-M., Chen H.-L., Lin Y.-X., Hang C.-H., Yin H.-X., Shi J.-X. (2009). N-Acetylcysteine Suppresses Oxidative Stress in Experimental Rats with Subarachnoid Hemorrhage. J. Clin. Neurosci..

[37] Mollica M.P., Trinchese G., Cavaliere G., De Filippo C., Cocca E., Gaita M., Della-Gatta A., Marano A., Mazzarella G., Bergamo P. (2014). C9,T11-Conjugated Linoleic Acid Ameliorates Steatosis by Modulating Mitochondrial Uncoupling and Nrf2 Pathway. J. Lipid Res..

[38] Cavaliere G., Trinchese G., Bergamo P., De Filippo C., Mattace Raso G., Gifuni G., Putti R., Moni B.H., Canani R.B., Meli R. (2016). Polyunsaturated Fatty Acids Attenuate Diet Induced Obesity and Insulin Resistance, Modulating Mitochondrial Respiratory Uncoupling in Rat Skeletal Muscle. PLoS ONE.

[39] Jaganjac M., Zarkovic N. (2022). Lipid Peroxidation Linking Diabetes and Cancer: The Importance of 4-Hydroxynonenal. Antioxid. Redox Signal..

[40] Javed A., Mehboob K., Rashid A., Majid A., Khan S., Baig Z.A. (2023). Oxidative Stress and Lipid Peroxidation in NAFLD with and without Type 2 Diabetes Mellitus. J. Coll. Phys. Surg. Pak..

[41] Inutsuka A., Yamanaka A. (2013). The Physiological Role of Orexin/Hypocretin Neurons in the Regulation of Sleep/Wakefulness and Neuroendocrine Functions. Front. Endocrinol..

[42] Monda V., La Marra M., Perrella R., Caviglia G., Iavarone A., Chieffi S., Messina G., Carotenuto M., Monda M., Messina A. (2017). Obesity and Brain Illness: From Cognitive and Psychological Evidences to Obesity Paradox. Diabetes Metab. Syndr. Obes..

[43] Tsujino N., Sakurai T. (2009). Orexin/Hypocretin: A Neuropeptide at the Interface of Sleep, Energy Homeostasis, and Reward System. Pharmacol. Rev..

[44] Messina A., De Fusco C., Monda V., Esposito M., Moscatelli F., Valenzano A., Carotenuto M., Viggiano E., Chieffi S., De Luca V. (2016). Role of the Orexin System on the Hypothalamus-Pituitary-Thyroid Axis. Front. Neural Circuits.

[45] Chieffi S., Carotenuto M., Monda V., Valenzano A., Villano I., Precenzano F., Tafuri D., Salerno M., Filippi N., Nuccio F. (2017). Orexin System: The Key for a Healthy Life. Front. Physiol..

[46] Hara T., Fujiwara H., Nakao H., Mimura T., Yoshikawa T., Fujimoto S. (2005). Body Composition Is Related to Increase in Plasma Adiponectin Levels Rather than Training in Young Obese Men. Eur. J. Appl. Physiol..

[47] Perez-Leighton C.E., Billington C.J., Kotz C.M. (2014). Orexin Modulation of Adipose Tissue. Biochim. Biophys. Acta (BBA)-Mol. Basis Dis..

[48] Tran L.T., Park S., Kim S.K., Lee J.S., Kim K.W., Kwon O. (2022). Hypothalamic Control of Energy Expenditure and Thermogenesis. Exp. Mol. Med..

[49] Gao Y., Sun T. (2016). Molecular Regulation of Hypothalamic Development and Physiological Functions. Mol. Neurobiol..

[50] Bétry C., Thobois S., Laville M., Disse E. (2018). Deep Brain Stimulation as a Therapeutic Option for Obesity: A Critical Review. Obes. Res. Clin. Pract..

[51] Ebrahim I.O., Howard R.S., Kopelman M.D., Sharief M.K., Williams A.J. (2002). The Hypocretin/Orexin System. J. R. Soc. Med..

[52] Shiraishi T., Oomura Y., Sasaki K., Wayner M.J. (2000). Effects of Leptin and Orexin-A on Food Intake and Feeding Related Hypothalamic Neurons. Physiol. Behav..

[53] Murphy K.G., Bloom S.R. (2006). Gut Hormones and the Regulation of Energy Homeostasis. Nature.

[54] Engel T., Goñi-Oliver P., Gomez-Ramos P., Morán M.A., Lucas J.J., Avila J., Hernández F. (2008). Hippocampal Neuronal Subpopulations Are Differentially Affected in Double Transgenic Mice Overexpressing Frontotemporal Dementia and Parkinsonism Linked to Chromosome 17 Tau and Glycogen Synthase Kinase-3β. Neuroscience.

